# Adaptive Governance of River Deltas Under Accelerating Environmental Change

**DOI:** 10.36633/ulr.803

**Published:** 2022

**Authors:** MANDY PAAUW, MURRAY SCOWN, ANNISA TRIYANTI, HAOMIAO DU, AHJOND GARMESTANI

**Affiliations:** Centre for Research on Environmental and Social Change (CRESC), University of Antwerp, BE; Copernicus Institute of Sustainable Development, Utrecht University and Lund University Centre for Sustainability Studies (LUCSUS), Lund University, NL; Copernicus Institute of Sustainable Development, Utrecht University, NL; Utrecht Centre for Water, Oceans and Sustainability Law (UCWOSL), Utrecht University, NL; Office for Research and Development, US Environmental Protection Agency, US

**Keywords:** Governance, adaptive governance, climate change, social-ecological systems, deltas

## Abstract

Many deltas are increasingly threatened by environmental change, including climate change-induced sea-level rise, land subsidence and reduced sediment delivery. Dealing with these challenges is a pressing necessity because deltas are home to many people and are important centres for economic and agricultural development. Successfully adapting to climate change requires a social-ecological system (SES) perspective, emphasising that social and ecological components of deltas are intertwined. Various modes of governance have been suggested to deal with uncertainty associated with environmental change in SESs, such as adaptive governance. Adaptive governance underlines the need for governance systems to be flexible enough to adapt to variable degrees of uncertainty in SESs. In this paper, we analyse the Dutch Delta Programme (DDP) and the Mekong Delta Plan (MDP) to explore their strengths and limitations relating to nine principles for adaptive governance proposed by DeCaro and others. We evaluate the suitability of this framework for the Rhine and Mekong deltas and contribute to the current understanding of delta governance in light of climate change. Most of the principles outlined by DeCaro and others are present in the DDP and MDP. However, adaptive governance is context dependent. The Rhine and Mekong deltas display different obstacles to adaptive governance, some of which are not sufficiently emphasised in this academic adaptive governance framework. Instead of relying on one framework as a blueprint for adaptive governance, using principles from different frameworks depending on the case may be the best approach for addressing environmental challenges in deltas.

## 1. INTRODUCTION

River deltas are highly dynamic systems. Under natural conditions, deltas evolve by the interaction of sediment deposition, redistribution and loss.^1^ Historical deposition of sediment in deltas has created fertile, low-lying coastal plains rich in biodiversity and in close proximity to abundant water resources, making them attractive for human settlement.^2^ Many deltas support growing cities and are important centres for agricultural production and economic development.^3^ More than 500 million people live in and around deltas today,^4^ illustrating that deltas are hotspots of social interactions with complex ecological and geomorphological processes.

The current rate of population and environmental change in deltas is higher compared with the global average.^5^ Populations are growing rapidly and urban areas are expanding into deltas. Concurrently, climate change is causing sea levels to rise,^6^ many major deltas are sinking,^7^ and sediment delivery to deltas is decreasing.^8^ These challenges are compounded by issues such as salinity intrusion and waterlogging.^9^ Although it is uncertain how these environmental challenges will unfold in the future, it is clear that delta drowning could result in the migration and displacement of millions of people, declining food security, biodiversity loss, ecosystem degradation, and loss and damage to the lives and livelihoods of delta inhabitants.^10^ Adapting to climate change and other environmental challenges in deltas is therefore a pressing and unavoidable necessity.^11^

Adaptation is not solely shaped by changing climatic and environmental conditions, it is determined by the responses of social systems to those changing conditions.^12^ For deltas to successfully adapt to environmental change, the interdependencies between social and environmental components of deltas must be recognised.^13^ This requires a social-ecological system (SES) approach to governance, emphasising that social and ecological systems in deltas can no longer be viewed independently but should be seen as strongly coupled.^14^ Various modes of governance have been put forward for dealing with uncertainty and complexity in dynamic SESs, such as adaptive governance,^15^ anticipatory governance,^16^ interactive governance,^17^ and transformative governance.^18^

An improved understanding of the governance of deltas is imperative in light of climate change, yet the number of studies that focus explicitly on governance in deltas is relatively limited.^19^ Here, we chose an adaptive governance framework because it focuses specifically on the need for governance systems to be flexible enough to adapt to variable degrees of uncertainty and complexity in SESs,^20^ which is highly relevant for deltas under accelerating environmental change. Furthermore, there is a need for comparative empirical case studies that apply principles for adaptive governance in different social and environmental governance contexts.^21^ Comparative approaches enable learning and knowledge sharing about the governance of deltas worldwide.^22^

In this paper, we study to what extent overarching delta management plans for the Rhine and Mekong deltas incorporate principles for adaptive governance. We evaluate the strengths and limitations of adaptive governance in the 2020 Dutch Delta Programme^23^ (DDP) and the 2013 Mekong Delta Plan^24^ (MDP) — policy documents that function as guidelines for dealing with environmental challenges in the deltas — by analysing them against principles for adaptive governance proposed by DeCaro and others.^25^ This analytical framework conceptualises the extent to which laws and institutions enhance conditions for self-organisation, flexibility and adaptation, and provides legal and institutional design principles for adaptive governance. The framework is highly suitable for assessing the DDP and MDP, because governing SESs such as deltas involves formal rules (laws) and stakeholders, but also other actors such as businesses, civil society organisations and citizens.^26^ Based on the application of the adaptive governance framework by DeCaro and others, we aim to assess the suitability of this framework for the Rhine and Mekong deltas specifically and contribute to a better understanding of the governance of deltas generally under climate change.

In Section 2 we discuss adaptive governance to illustrate the applicability of this perspective for the governance of SESs such as deltas. Section 3 introduces the analytical framework used. This is followed in Section 4 by an outline of the methods used for the analysis of the DDP and MDP. The results are presented in Section 5. In Section 6, we reflect on lessons learned from applying an adaptive governance perspective to the Rhine and Mekong cases. We conclude, in Section 7, with recommendations for governance of deltas under accelerating environmental change and ideas for future research.

## 2. ADAPTIVE GOVERNANCE

One of the most persistent obstacles to the sustainable management of deltas relates to governance.^27^ The governance of deltas is complex due to the often international character of their river basins and changing nature of both the ecological and social dimensions.^28^ Deltas are dynamic SESs characterised by uncertainty and unpredictability. Top-down, centralised governance is not ideal for dealing with uncertainty and unpredictability in SESs, because it often treats SESs as if they are linear, predictable, or even unchanging, resulting in rigid policies and a lack of adaptability.^29^ Effectively governing deltas requires a shift away from a steady-state view that aims to control and stabilise SESs, assuming gradual changes as the norm and ignoring interactions within and across systems and scales.^30^

Adaptive governance has been put forward as an alternative governance approach to deal with the uncertainty and unpredictability inherent in SESs.^31^ Adaptive governance emphasises the need for governance systems to be flexible enough to adapt to feedback from the social and ecological parts of the system it governs.^32^ Complex problems such as climate change, land subsidence and reduced sediment delivery in deltas embed different interests and perspectives, which have to be considered to generate suitable governance.^33^ By embracing a broad set of actors, organisations and institutions, adaptive governance aims to create polycentric, multilevel institutions where power and responsibility are shared among government agencies and non-governmental organisations, creating flexible and responsive governance conditions.^34^ Including a variety of stakeholders can stimulate collaboration and trust building,^35^ whilst also giving access to different kinds of knowledge vital for social learning and innovation.^36^ Learning and experimentation are integral to the adaptive capacity of governance systems, because they allow actors to improve their understanding of the SES and adapt their behaviour accordingly.^37^ However, without leadership showing direction and motivating others, governance systems may be unable to respond to challenges that affect society.^38^

Adaptive governance underlines the importance of managing resilience in SESs to address uncertainty and surprises.^39^ Resilience refers to the ability of SESs to change and persist whilst maintaining the same processes and structures (i.e., remain in their current regime).^40^ Adaptive governance builds on theories of resilience and aims to incorporate feedbacks from changing SESs before they shift into an undesirable state.^41^ This is important for the long-term sustainability of SESs, if the governance plan revolves around perpetuating the current configuration;^42^ in other words, if the current state of the SES is still acceptable from a biophysical and stakeholder perspective. Transformative governance has similar factors to adaptive governance, but requires more (e.g., risk tolerance, monetary investment)^43^ to push a SES (with human agency) to a new configuration. If conditions in the SES are too degraded, for example, the catastrophic decline of coal mining jobs in West Virginia in the United States, then transformation via transformative governance to a new regime with a different set of processes and structures in the SES (e.g., eco-tourism, organic farming, solar panel manufacturing) is a more appropriate course of action.^44^

## 3. ANALYTICAL FRAMEWORK

The adaptive governance framework by DeCaro and others provides five legal (e.g., written enforceable rules) and four institutional (e.g., locally accepted norms) design principles for adaptive governance. The legal design principles concern ‘(a) elements of official legal systems that determine structure, authority, function, and guidelines for government agencies […] and private centres of activity (e.g., individuals, industry, grassroots organisations) and (b) rules and regulatory systems that deal with compliance’.^45^ Legal design principles in the framework include reflexive law, legal sunsets, legally binding authority, legally binding responsibility and tangible support. The institutional design principles ‘refer more broadly to features of rule-governed systems, like clearly defined socio-political and geographic boundaries, that help to solve problems collectively’.^46^ Institutional design principles in the framework include well-defined boundaries, participatory decision-making, internal enforcement and internal conflict resolution. An overview of the principles, as well as their definition and associated key concepts, can be found in Table 1 below.

The legal and institutional design principles create conditions for the emergence of adaptive governance within society. Principles such as participatory decision-making, legally binding authority and responsibility aim to create favourable conditions for the emergence of partnerships and compacts between stakeholders.^47^ This is crucial for adaptive governance, because the inclusion of different stakeholders enhances conditions for flexibility, creativity, innovation and responsiveness needed to adapt governance systems to changes in a SES.^48^ Internal conflict resolution and internal enforcement generate conditions for fairness and legitimacy, essential to cultivate trust among the participating stakeholders. Technical, financial and informational support provide public and private stakeholders with sufficient resources to successfully implement adaptive measures;^49^ while principles such as reflexive law and legal sunsets stimulate decision-making that is flexible, iterative and open to revision, encouraging learning required to deal with the uncertain, complex and ever-changing nature of SESs.^50^

## 4. METHODOLOGY

### 4.1. RESEARCH MATERIALS

We conducted a content analysis to explore the strengths and limitations of the 2020 Dutch Delta Programme (DDP) and the 2013 Mekong Delta Plan (MDP) by analysing them against principles for adaptive governance formulated by DeCaro and others. Content analyses are useful research tools to determine the presence of, e.g., principles within qualitative data, and to analyse the meanings of these principles. The DDP and MDP are policy documents that function as guidelines for dealing with climate change, sea-level rise and other environmental challenges such as subsidence, underlining the relevance of these policy documents for this analysis. Comparing the Rhine and Mekong deltas also provides insights from different global north-south contexts. In subsections 4.1.1–4.1.3 we provide some general information about the Rhine and Mekong deltas as well as an explanation of the content of the delta plans.

#### 4.1.1. The Rhine delta and the DDP

The Rhine-Meuse-Scheldt delta (in short, the Rhine delta) is located in the south-eastern corner of the North Sea Basin. The low-lying delta plain is 25,347 km^2^, making it the largest delta in Europe.^51^ The majority of the delta is located in the Netherlands. Most of the Dutch population is concentrated in the coastal lowlands of the Rhine delta^52^ and the area is of great economic importance with 65% of the Dutch gross national product being generated here. Protecting the delta’s inhabitants, their livelihoods, infrastructure, etc. is therefore of critical importance, which explains why the Rhine delta is heavily engineered and shaped by human interventions.^53^ Most of the damming of the delta was initiated by destructive floods in 1953, which also led to the formulation of the first DDP committed to improving flood protection systems in the Netherlands.^54^ Although the increasing number of dams and embankments reduced the occurrence of floods, it also led to a number of environmental challenges. Reduced sediment deposition on the delta plain, combined with drainage and land reclamation, resulted in land subsidence.^55^ This is compounded by sea-level rise, which increases the delta’s vulnerability to flooding and storm surges, saltwater intrusion and increases the risk of permanent inundation. Furthermore, there is no guarantee that embankments will be sufficient to protect the Rhine delta and its inhabitants from sea-level rise.^56^

The DDP is a policy document from the Dutch government to protect the Netherlands against increasing flood risks resulting from climate change. It is an initiative in which the Dutch government works together with provinces, municipalities, regional water authorities, civil society organisations, research institutes, businesses and citizens.^57^ The DDP establishes and reports on the progress on five Delta Decisions,^58^ which contain plans to protect the Netherlands from flooding and water shortages. The Delta Decisions form the basis of Dutch water policy,^59^ and are anchored in the National Water Plan,^60^ which describes the general aim and direction of water policy in the Netherlands, in the Water Act^61^ and in administrative agreements with local governments. Delta Decisions provide long-term, strategic policy objectives, to be further developed and implemented in relevant policy domains. The DDP is structured around three main themes: water safety (mainly flood protection and flood risk management), freshwater and spatial adaptation. The measures proposed in the DDP are mostly financed through the Delta Fund. Every year, the Delta Commissioner presents the new DDP to the Minister of Infrastructure and Water Management, who is ultimately responsible for the development of and progress on the DDP. The Delta Commissioner promotes the implementation of the DDP and monitors its progress.^62^

#### 4.1.2. The Mekong delta and the MDP

The low-lying Mekong delta is approximately 62,520 km^2^, of which 52,100 km^2^ are located in Vietnam.^63^ The Mekong delta is the largest delta in Southeast Asia and the third largest delta in the world, home to about 20 million people with a population density of up to 500 persons per km^2^.^64^ The area is of great economic importance. It provides 50% of Vietnam’s food production and more than 200 million people rely on the delta for food.^65^ However, anthropogenic activities are threatening the sustainability of the Mekong delta. The construction of dams in upstream countries reduces sediment supply to the delta, exacerbating delta shoreline erosion.^66^ Embankments have been constructed to deal with increasing flood risks and the destabilising of channel banks, but these changes have their own negative impacts such as increasing river flow velocities causing flood hazards and erosion.^67^ Furthermore, the Mekong delta is sinking rapidly, which, combined with sea-level rise, increases the delta’s vulnerability to flooding and storm surges, saltwater intrusion, salinisation of groundwater, coastal erosion and inundation.^68^ This significantly threatens the lives and livelihoods of the delta’s inhabitants as well as its agricultural productivity.

To deal with these environmental challenges, the MDP was created together by the Socialist Republic of Vietnam and the Kingdom of the Netherlands under the Strategic Partnership Arrangement on Climate Change Adaptation and Water Management Arrangement.^69^ The MDP aims to respond to the consequences of climate change and to ensure sustainable socioeconomic development of the Mekong delta. In Vietnam, partners in developing the MDP included the Ministry of Natural Resources and Environment and the Ministry of Agriculture and Rural Development. Dutch partners included the Dutch government, Wageningen University, Deltares (a knowledge institute), Rebel (a management consultancy organisation), and Royal HaskoningDHV and Water.nl (consultancy firms). The MDP is presented as a reference document for Vietnamese government agencies and organisations at all levels. It functions as a tool to support the review, coordination and integration of current policies for the delta as well as a guideline for the implementation of these policies.^70^

#### 4.1.3. Similarities and differences between the DDP and MDP

The DDP and MDP are similar in that they both describe and propose strategies to protect the deltas against flooding. The documents provide guidelines to deal with uncertainty regarding climate change and other environmental challenges such as reduced sediment delivery. However, the documents differ in their legal basis. The formulation of the DDP is prescribed in the Dutch Water Act^71^ and Delta Act.^72^ The Water Act brings together a range of laws on water management and flood protection. It is legally defined that a DDP must be published every year. The Minister of Infrastructure and Water Management presents the DDP to the Parliament together with the budget for the Delta Fund for the new year.^73^ The MDP, on the other hand, has no formal status in the Vietnamese legal system but rather provides strategic advice to the government of Vietnam.^74^ However, the principles and strategies proposed in the MDP were embraced by Government Resolution 120, which provides policy guidance on ‘Sustainable and Climate-Resilient Development of the Mekong Delta of Vietnam’.^75^ An action plan to further implement Resolution 120 was issued in 2019 with Decision 417.^76^ In addition, a new plan for the sustainable development of the Mekong delta has recently been launched: the Mekong Delta Regional Master Plan,^77^ which can be seen as a successor to the MDP.

### 4.2. METHOD OF DOCUMENT ANALYSES

For each of the nine design principles provided in the adaptive governance framework by DeCaro and others, key concepts were identified which were used to assess the DPP and MDP (Table 1). Some of the key concepts were taken directly from the DeCaro and others’ framework, others were formulated based on the principles’ descriptions. After the key concepts were identified, each of the design principles was assigned its own colour. The DDP and the MDP were colour coded by highlighting text referring to the key concepts presented in Table 1.

The principles that were found to be present in the DDP and the MDP were further assessed based on their clarity and frequency. Clarity refers to whether a principle is mentioned explicitly or implicitly in the documents. If the documents specifically mentioned the key concepts associated with a principle, that principle was assumed to be explicitly present for this analysis. If the documents referred to a principle without mentioning the associated key concepts, that principle was assumed to be implicitly present for this analysis (e.g., the documents refer to a principle using terms or concepts that differ from the key concepts identified in Table 1, or the documents refer to other sources where the principles are explicitly present). Hence, we assume that even if a principle is implicitly present, this can still create conditions for adaptive governance.

After colour coding the DDP and the MDP, the highlighted pieces of text were counted providing an indication of the frequency of mentions of the principles in the documents. Some principles were mentioned only a few times (< five times) while others were mentioned frequently (> five times). We interpret the frequency of mentions of a principle to reflect its perceived importance by contributors to the DDP and MDP and potentially to decision-makers using the plan. However, we acknowledge that, from a legal perspective, a single stipulation of any obligation can provide strength and binding effect.

## 5. ADAPTIVE GOVERNANCE IN THE DUTCH DELTA PROGRAMME AND THE MEKONG DELTA PLAN

**Figure 1:**
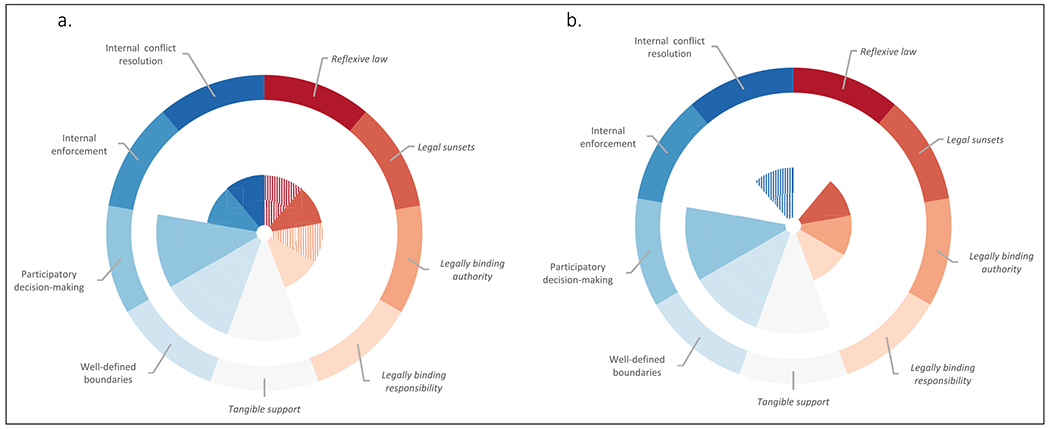
Overview of the results of the DDP **(a)** and MDP **(b)** analyses. Radiating bars indicate the frequency with which the legal (in *italics* and red shades) and institutional design principles (in normal text and blue shades) for adaptive governance were present in the documents. The absence of bars indicates the absence of a principle, short bars indicate a low frequency (< five times) and long bars indicate a high frequency (> five times). Principles explicitly present in the documents are represented by uniform-coloured bars whereas principles implicitly present are represented by striped bars.

In general, there is good coverage of adaptive governance principles in the DDP and MDP. Most of the adaptive governance principles presented by DeCaro and others are present in the documents, to varying degrees of clarity and frequency (Figure 1; Table 2). The DDP includes all design principles. The MDP includes four out of five legal design principles and three out of four institutional design principles.

### 5.1. STRENGTHS OF THE DDP AND MDP REGARDING ADAPTIVE GOVERNANCE

The DDP and MDP score well on three of the nine adaptive governance principles provided by DeCaro and others. Both delta plans emphasise tangible support, well-defined boundaries and participatory decision-making. These principles are explicitly present and mentioned frequently in the documents (Figure 1). We argue that the emphasis on these three principles is a strength of the DDP and MDP that may contribute to the emergence of adaptive governance in the deltas.

#### 5.1.1. Tangible support

Examples of tangible support for stakeholders are frequently provided throughout the DDP and MDP. Tangible support underlines that stakeholders need proper support in the form of, e.g., funds, technology, information, or training from central and local governments to successfully participate in the design and implementation of decisions.^100^ Some examples of tangible support in the DDP and MDP are provided below.

The DDP explicitly states that the Delta Programme is financially supported by the Delta Fund. ‘The Delta Fund holds the financial resources to finance investments in water safety, freshwater and water quality, and the central government’s management and maintenance activities that pertain to this.’^101^ Due to an amendment to the Water Act, which came into effect on 1 January 2021, financial contributions from the Delta Fund can now also be granted to decentralised authorities, to aid them in implementing measures against coastal, fluvial and pluvial flooding.^102^ Furthermore, the Dutch Minister for Infrastructure and Water Management made extra funds available for stimulating and facilitating climate change adaptation, meant for pilot projects, knowledge development and knowledge sharing through an online platform.^103^ The knowledge platform supports the Dutch National Climate Adaptation Strategy and the plan on spatial adaptation in the DDP, and provides information to (local) governments, businesses and civil society organisations to aid in planning for a climate-proof, water-resilient future.^104^ In the MDP, an example of tangible support in the form of funds can be found in the proposed flood management measures for the delta. For each of these measures, it is clearly indicated how the measures can be financially supported by the central government.^105^ Furthermore, the MDP underlines the importance of support in the form of information. The MDP stipulates that the central government should produce a joint knowledge agenda to increase the availability and accessibility of data and information for all stakeholders in the Mekong delta, to aid them in participating in the implementation of measures for a sustainable Mekong delta.^106^

#### 5.1.2. Well-defined boundaries

According to the principle of well-defined boundaries, socio-political and ecosystem boundaries of an environmental dilemma should be recognised and well-defined.^107^ The DDP and MDP provide multiple examples of agreements about socio-political or ecosystem boundaries that support the principle of well-defined boundaries.

The DDP clearly acknowledges the international character of rivers and that measures taken in the upstream river will impact the lower delta and vice versa. The Rhine, Meuse and Scheldt rivers all cross international borders, as well as the borders of water management authorities in the Netherlands. This requires collaboration between different administrative entities within the Netherlands and with international stakeholders. For example, the DDP refers to the Policy Platform Water Safety, in which Dutch stakeholders collaborate on water safety issues, including the central government, provincial and local governments, and regional water authorities.^108^ In addition, various international collaborations are mentioned in the document, such as the International Commission for the Protection of the Rhine, the Working Group High Water (for the protection of the Lower Rhine), the International Scheldt Commission (for the sustainable management of the Scheldt river) and the Flemish-Dutch Scheldt Commission (for the sustainable management of the Scheldt estuary).^109^ Similarly, the MDP underlines that ‘arrangements for flood control, securing of adequate freshwater supplies in the dry season, salinity intrusion, regulation and management of an adequate and healthy brackish water zone for aquaculture, coastal defence, etc. are all typically measures that need to be considered at the delta level, [but] in their impact and influences they go beyond the boundaries of local governance and policy jurisdiction’.^110^ Towards that end, the MDP distinguishes between the upper, middle and lower reaches of the delta and recognises that different parts of the delta need different strategies. The development and implementation of these strategies requires stakeholder collaboration.

#### 5.1.3. Participatory decision-making

Participatory decision-making prescribes that affected local and regional stakeholders should be able to influence the design and implementation of decisions.^111^ The DDP and MDP provide numerous illustrations of processes and methods that enable stakeholder participation. For example, the DDP is the product of the collaboration between the central government, provinces, municipalities and regional water authorities, and the Programme is based on ‘input from civil society organisations, knowledge institutes, citizens, and businesses’.^112^ The DDP also distinguishes between five levels of participation from which the appropriate level can be chosen depending on the specific project. This participation ladder includes ‘informing, consulting, advising, co-producing and (co-)decision-making’.^113^ Similarly, various knowledge institutes were included in the preparatory phase of establishing the MDP. Stakeholders are also consulted in the implementation of the proposed strategies in the MDP, including ‘experts and specialists from different sectors with a bird’s-eye view across the sectors, decision-makers of local, provincial, and national authorities, [and] representatives from organisations for e.g., industry, fishery, transport, agri- and aquaculture’.^114^

### 5.2. ABSENCE OF PRINCIPLES FROM THE DDP AND MDP

All adaptive governance principles from the framework by DeCaro and others are present in the DDP. In the MDP, on the other hand, two principles are currently absent: the legal design principle of reflexive law and the institutional design principle of internal enforcement (Figure 1; Table 2).

The definition of reflexive law provided by DeCaro and others prescribes that laws should define procedural norms and establish ground rules, instead of focussing on specific outcomes as the ultimate result.^115^ By emphasising standards and general principles, decision-makers at local levels of government have legal guidance but also flexibility when they need to decide on local issues. The MDP does not refer to reflexive principles. This is a limitation for the emergence of adaptive governance in the Mekong delta, because reflexive principles allow for flexible decision-making at lower levels of government needed to deal with complex and multi-scale SES dynamics.^116^

The principle of internal enforcement is also missing from the MDP. Stakeholder participation creates conditions for creativity, innovation and knowledge sharing which is essential for flexibly governing SESs.^117^ However, internal enforcement is needed to monitor the behaviour of participating stakeholders in the governance of SESs and enforce their compliance with rules.^118^ Monitoring also makes those who do not comply with rules visible to the community, which, in turn, increases the effectiveness of rule enforcement mechanisms.^119^ Currently, the MDP does not provide illustrations of mechanisms to monitor the behaviour of participating stakeholders and enforce their compliance, a second limitation for the emergence of adaptive governance in the Mekong delta.

## 6. DISCUSSION

### 6.1. STRENGTHS AND WEAKNESSES OF THE DDP AND MDP

The analyses of the DDP and MDP reveal strengths of these delta plans along the same three adaptive governance principles: tangible support, well-defined boundaries and participatory decision-making.

Participatory decision-making is essential for the emergence of adaptive governance. Different stakeholders have access to different types of information, knowledge and expertise, creating innovative and creative governance conditions needed to deal with the complex nature of deltas.^120^ Furthermore, stakeholder participation can enhance flexibility and responsiveness in decision-making. Decentralising decision-making authority and responsibility can stimulate fast responses to changing local conditions^121^ (e.g., flash floods), because local and regional stakeholders have easy access to information about local issues and are the first to know when conditions change.^122^ This is highly relevant for deltas, as governance systems have to deal with a multitude of uncertainties. Participatory decision-making also underlines the importance of stakeholder participation for consensus building and long-term social learning in the governance of dynamic SES^123^ which can contribute to the sustainability of the Rhine and Mekong deltas.

However, the participation of local governments, businesses, civil society organisations and citizens in the governance of deltas can be difficult without adequate and sufficient resources.^124^ The emphasis of the DDP and MDP on tangible support would ensure that stakeholders receive adequate (financial, informational, technological) support to participate in decision-making processes and implementation of strategies. Tangible support may also stimulate experimental processes as stakeholders are incentivised to implement their own strategies. Implementation of a variety of strategies by diverse stakeholders may contribute to social learning about the SES being governed and is a central aspect of adaptive governance.^125^ Without sufficient support, stakeholders cannot perform their tasks satisfactorily,^126^ which can result in adaptive and cooperative failures.^127^ Furthermore, the strengths of the DDP and MDP also lie in the emphasis on well-defined boundaries. Working together with a variety of local, regional, national and international stakeholders to govern complex SESs such as deltas can be confusing without a clarification of the jurisdictions of these stakeholders. Well-defined socio-political and ecosystem boundaries facilitate collaboration between different stakeholders and collective problem solving.^128^

The similarities in strengths in the DDP and MDP are not surprising, given that the MDP was created through a collaboration between the Vietnamese and Dutch governments. While we argue these are strengths potentially enabling and encouraging adaptive governance to deal with accelerating and uncertain environmental change, transfer of knowledge and policy from the Rhine delta to the Mekong delta should be done with caution given the contextual differences in environment, culture, politics, law and economics between these deltas. Take, for example, participatory decision-making: water governance in the Netherlands traditionally was based on cooperation and consensus building among stakeholders, called the polder model.^129^ During the Middle Ages, many low-lying areas were reclaimed from bodies of water and subsequently protected by dikes against flooding. Windmills were used to pump water from the polders. Maintaining this system required cooperation between a variety of stakeholders.^130^ Therefore, the principle of participatory decision-making is arguably rooted in Dutch tradition and culture. However, such cultural roots may not be mirrored in Vietnam. The principles of tangible support and well-defined boundaries, which are also emphasised in the two plans, are arguably related to stakeholder participation. Tangible support underlines that stakeholders need sufficient resources to participate, and well-defined boundaries are needed to clarify the jurisdictions of these stakeholders, facilitating cooperation and collective problem solving.^131^ However, the amount of support available and the jurisdictions of stakeholders are likely to differ substantially between the Rhine and Mekong deltas.

In general, the legal dimensions of the adaptive governance framework by DeCaro and others are somewhat underrepresented in the DDP and MDP (Figure 1). Although all legal design principles are present in the DDP, they are infrequently present and two of them (reflexive law, legally binding authority) are only implicitly mentioned. Legal design principles in the Netherlands are mainly rooted in formal legislation and are therefore not stipulated in policy documents such as the DDP. The same is true for the MDP. The MDP does, however, provide explicit references to legal principles such as legally binding authority and legally binding responsibility. For example, the MDP refers to the Law on Water Resources, one of the most important laws for the governance of water resources, in particular allocating the rights and duties of different stakeholders. However, such references are sparse in the MDP as authorities and responsibilities are normally stipulated in formal legislation. The underrepresentation of legal design principles in the DDP and MDP can be explained by the fact that the policies function as guiding documents supporting governmental and non-governmental stakeholders in achieving a shared goal: the sustainable development of the Rhine and Mekong deltas respectively. The DDP and MDP set out general, strategic visions to achieve that goal. We acknowledge that the frequency or clarity of legal design principles in the DDP and MDP does not determine the legal space for the emergence of adaptive governance in the Rhine and Mekong deltas. In this paper, we provided an introductory analysis of overarching delta management plans to understand enabling conditions for the emergence of adaptive governance in deltas. Although it is beyond the scope of this paper to analyse relevant laws and regulations in addition to the DDP and MDP, we recommend a systematic approach that assesses policy plans, institutional documents and legal instruments for future research.

### 6.2. REFLECTION ON THE ADAPTIVE GOVERNANCE FRAMEWORK BY DECARO AND OTHERS

Adaptive governance is a useful framework for dealing with the complexity and uncertainty resulting from accelerating environmental change in SESs.^132^ Successful adaptation in SESs often emerges from collaborative, creative processes initiated by governmental and nongovernmental stakeholders.^133^ The adaptive governance framework by DeCaro and others used in this paper focuses specifically on aspects of laws and institutions that support governing for resilience in SESs. A considerable strength of the framework is that it provides guidance for legal and institutional design that can foster the emergence of adaptive governance. The framework also allows for comparing different cases which may enable learning and knowledge sharing about adaptive governance in different locations.

However, adaptive governance is context dependent. It is often not the same in two places because it develops within the context of a specific SES.^134^ DeCaro and others underline this and emphasise that, although supporting more of the legal and institutional design principles can lead to the emergence of adaptive governance, the framework is a working hypothesis. It is crucial, according to the authors, to investigate how the ideas from the framework translate to local cases.^135^ From the application of the framework to the Rhine and Mekong cases, we found that both deltas experience different obstacles to the emergence of adaptive forms of governance and require an emphasis on different adaptive governance principles. We further elaborate on these obstacles for both deltas in the paragraphs below.

The DDP emphasises the principle of participatory decision-making. As already mentioned, this could be explained by the fact that water governance in the Netherlands traditionally was a shared responsibility of various stakeholders. Legally binding authority and responsibility are also present in the DDP, indicating that stakeholders have the authority to make decisions, implement chosen solutions, and are held responsible for their actions (or inaction). However, today, water management in the Netherlands remains mainly a state task as flood risks are so high that they are considered a vital threat to the habitability of the country.^136^ Participation of the public in agenda-setting, decision-making, implementation and evaluation of flood measures^137^ remains limited. In the Netherlands, citizen involvement generally occurs in the form of consultation instead of partnerships.^138^ This contrasts with other European countries such as the UK and Belgium where flood risks are increasingly considered as a shared responsibility between water managers, other governmental actors and citizens.^139^

Fliervoet and others^140^ demonstrate through a social network analysis that many nongovernmental actors in the Netherlands are not seen as equal partners in the governance of water resources. Non-governmental actors remain highly dependent on the main governmental organisation which obstructs the shift from a dominant government towards collaborative, polycentric forms of governance in the Netherlands. Polycentric systems are a key component of adaptive governance and are defined as complex, adaptive systems without a central authority dominating the processes and structures of the system.^141^ For adaptive governance to emerge in the Netherlands, governmental organisations need to recognise non-governmental actors as equal partners.^142^ With equal partnerships, we mean that stakeholders do not stand in a hierarchical relationship to each other.^143^ This may result in responsive and flexible governance systems capable of dealing with increasing levels of change and uncertainty.^144^ Individual or community-based actions, which could be considered as an addition to public flood defence, are often small-scale measures which can be implemented and revised relatively quickly,^145^ and would therefore contribute to the adaptive capacity of the Rhine delta. Individual or community-based actions could be stimulated by public authorities by allocating adequate resources to water management partners, such as financial support targeted at the most vulnerable groups.^146^

Ha and others^147^ found multiple obstacles to the emergence of adaptive governance in the Mekong delta, some of which are also addressed in the framework by DeCaro and others. For example, they found limitations in vertical and horizontal integration, which is also the case in the Rhine delta,^148^ and in public participation. Vertical integration refers to a well-balanced distribution of responsibilities and authorities and the involvement of lower-level actors in higher levels of decision-making.^149^ This is addressed in the adaptive governance framework by DeCaro and others with the principles of legally binding authority and responsibility. Ha and others also found limitations in public participation, which refers to provisions that support the participation of stakeholders in the formulation of rules that affect them,^150^ which aligns with the principle of participatory decision-making in the framework used in this paper. The overlap between some of the obstacles identified by Ha and others with the design principles for adaptive governance in the framework by DeCaro and others highlights the relevance of the design principles for the Mekong delta. To overcome the obstacles in vertical and horizontal integration and public participation,^151^ the principles of legally binding responsibility and authority, and participatory decision-making, which are present in the MDP, should be more clearly and concretely implemented in Mekong delta governance.

Ha and others also identified factors that obstruct the emergence of adaptive governance in the Mekong delta that are not sufficiently emphasised in the framework by DeCaro and others used in this paper. For example, lack of small-scale policy experimentation is identified as an obstacle to adaptive governance in the Mekong delta.^152^ Considering policies and management actions as small-scale experiments is important for adaptive governance, because it provides information about how the system responds to policy and management actions, and thus provides an opportunity to learn about the functioning of a SES.^153^ It could be argued that policy experimentation is a type of reflexive component, emphasised by DeCaro and others through the principle of reflexive law. However, the definition of this principle by DeCaro and others does not include experimentation specifically, which may be an oversight that is slightly detrimental to the framework.

Another barrier to the implementation of adaptive governance in the Mekong delta relates to knowledge sharing. Ha and others found that knowledge is not proactively shared by the government with the public.^154^ This is problematic. Active knowledge sharing can help empower social groups to participate in governmental decision-making processes.^155^ Although the framework by DeCaro and others discusses the importance of tangible support for stakeholders in the form of funds, technology, information, or training, a focus on active knowledge and information management is absent. Important for the emergence of adaptive governance in the Mekong delta is collaborative knowledge production, diversity in knowledge, and knowledge and information sharing.^156^

### 6.3. LESSONS LEARNED FROM APPLYING AN ADAPTIVE GOVERNANCE PERSPECTIVE

The above discussion shows that adaptive governance faces different obstacles in the Rhine and Mekong deltas, underlining that adaptive governance is context dependent. Assuming a set of fixed design principles for adaptive governance and applying them to different deltas worldwide is difficult, because it prevents us from seeing the full range of governance problems and potential solutions. Enabling conditions for adaptive governance are context-specific and design principles need to be adapted depending on the local situation. This is important, because governance of climate change and environmental challenges is more likely to be successful when the governance system is adjusted to the SES it tries to manage.^157^ Hence, the adaptive governance principles by DeCaro and others should be used as a generalised guideline and if principles do not directly translate to a particular SES, they should be adapted to the case at hand. Furthermore, other principles that enable adaptive forms of governance, such as those identified by Ha and others for the Mekong delta, should perhaps be added for particular contexts.

### 6.4. LIMITATIONS OF METHODS USED

The methods employed in this paper have some limitations which we briefly discuss here. We acknowledge that we analysed a limited number of policies: the 2020 DDP and the 2013 MDP. In addition, the DDP is an ongoing development. It is updated yearly and each DDP builds upon the previous year. We chose the DDP from 2020 because this was the most recent DDP at the time of the analysis. The results in this paper may have been slightly different if the analysis were to be applied to a more recent DDP, e.g., the 2022 DDP, as currently more information and knowledge may be available on the progress and pace of environmental change, as well as on how to deal with these challenges in an adaptive way. However, it is unlikely that the 2022 DDP will differ significantly from the 2020 DDP, as the policy changes incrementally over time. To fully respect the gradually evolving and iterative nature of the DDP, future research should analyse a series of DDPs. However, systematic reassessments of the DDP do not take place yearly but once every six years. Hence, we expect minor differences between the analysis of the 2020 DDP and those published thereafter. The MDP, in contrast to the DDP, was published once, in 2013, and functions as a guideline for the Vietnamese government.

Another limitation of the work is that the framework used in this study provides legal and institutional design principles for adaptive governance; however the DDP and MDP are policy documents. This has impacted our results as the legal design principles offered by DeCaro and others are somewhat underrepresented in the DDP and MDP. As mentioned, we acknowledge that the frequency or clarity of the legal design principles in the DDP and MDP do not determine the legal space for adaptive governance. Rather, in this paper we aim to provide an introductory analysis of overarching delta management plans to understand enabling conditions for the emergence of adaptive governance in deltas. However, for future research, we recommend a systematic approach that assesses policy plans, institutional documents and legal instruments, as well as stakeholder interviews and surveys, to better capture the governance context in the Rhine and Mekong deltas. Finally, it is also important to consider that we compared a developed country (Netherlands) with a developing country (Vietnam).

## 7. CONCLUSION

Adaptive governance has been put forward as a framework for addressing uncertainty and unpredictability in complex SESs such as deltas. We applied an adaptive governance framework to overarching delta management plans for the Rhine and Mekong deltas, the DDP and MDP respectively, demonstrating that most of the design principles for adaptive governance offered by DeCaro and others are present. The strengths of both delta plans regarding adaptive governance lie in the emphasis on tangible support, well-defined boundaries and participatory decision-making. However, the application of the framework to the Rhine and Mekong deltas also shows that governance is likely to need to be adapted to the local context. In both deltas, there are case-specific conditions that obstruct the emergence of adaptive governance, conditions which may be insufficiently covered in the framework by DeCaro and others. Adaptive governance of SESs is often not the same in two places. Therefore, governance principles such as those suggested by DeCaro and others should be seen as a guideline and modified depending on the local situation.

Our understanding of the governance of deltas under accelerating environmental change is still limited. As governance systems are extremely important in generating adaptive capacity of a region to environmental change, we recommend systematic, detailed analyses of legal and governance structures in the Rhine and Mekong deltas, but also in other deltas across the globe.^158^ Detailed analyses of legal and governance structures is important to improve our understanding of the enabling and constraining conditions for adaptive governance of deltas.^159^ In addition, future research should consider the limitations of this study and account for them in further studies, e.g., by incorporating a variety of legal and institutional documents, as well as further research on transformative governance.

## Figures and Tables

**Table 1:** Legal and institutional design principles for adaptive governance by DeCaro and others. The second column provides a description of what the principles entail; the third column shows an operationalisation of the principles in the form of key concepts used to assess the DDP and MDP.

LEGAL DESIGN PRINCIPLES	DEFINITION	KEY CONCEPTS USED TO ASSESS DELTA PLANS
1. Reflexive law	Laws should not rely on static rules when flexibility is needed; legal systems need to emphasise standards and general principles instead of specific rules about final solutions so that decision-makers have legal guidance but also flexibility when they need to make decisions	Minimum requirements (floors), maximum thresholds (ceilings), general guidelines (principles)
2. Legal sunsets	Laws include planned periods of evaluation in which environmental policies and agreements can be re-examined, renegotiated and modified if needed; this allows for safeguarding security and stability without jeopardising flexibility	Incremental revisions after specific time-periods, planned windows of opportunity, distinction between short-and long-term measures
3. Legally binding authority	The authority of stakeholders to make decisions, implement solutions and carry out plans is institutionalised in binding legislation, to ensure decision-making latitude for stakeholders	Laws or formal rules legitimising decision-making latitude for stakeholders
4. Legally binding responsibility	The devolution of responsibility to resolve or contribute to a resolution or dilemma needs to be formally defined and assigned, to motivate stakeholders to help resolve environmental dilemmas	Laws or formal rules defining and assigning responsibility
5. Tangible support	Devolution of responsibility may be overwhelming without technical and financial support; for stakeholders to meet their responsibilities and pursue their authority, support from the central and local governments is required	Support in the form of funds, technology, information, or training
INSTITUTIONAL DESIGN PRINCIPLES	DEFINITION	KEY CONCEPTS USED TO ASSESS DELTA PLANS
1. Well-defined boundaries	Socio-political and ecosystem boundaries of environmental dilemmas are well-defined, which aids in clarifying the legal and institutional jurisdiction of stakeholders	Compacts or agreements about socio-political and ecosystem boundaries
2. Participatory decision-making	Affected stakeholders can influence the design and implementation of strategies through participatory decision-making, which allows for the inclusion of a variety of stakeholders	Processes or methods enabling and stimulating stakeholder participation
3. Internal enforcement	Organisations and collectives have internal mechanisms to monitor and enforce compliance, in addition to external monitoring, enforcement and graduated sanctioning to safeguard rules	Monitoring mechanisms such as periodic check-ups or mandatory progress reporting, and enforcement mechanisms such as financial incentives
4. Internal conflict resolution	Internal mechanisms for neutral and transparent conflict resolution	Communication, internal ‘quasi-formal’ courts to resolve disputes

**Table 2:** Overview of the adaptive governance principles by DeCaro and others present in the DDP and MDP, including examples illustrating how the principles are formulated in the documents. This is not an exhaustive list of all references to the principles that can be found in the delta plans.

LEGAL DESIGN PRINCIPLES	DUTCH DELTA PROGRAMME (DDP)	MEKONG DELTA PLAN (MDP)
1. Reflexive law	Reflexive law is implicitly present in the DDP. The DDP distinguishes between broad Delta Decisions, region-specific preferred strategies, and local projects. Delta Decisions provide a general vision for the future of the Rhine delta, preferred strategies are region-specific applications of Delta Decisions and projects tackle issues at the local level. In other words, Delta Decisions provide overall goals and general guidelines whereas preferred strategies and projects fill in detailed policy lines. This aligns with the principle of reflexive law, although key concepts were not specifically mentioned in the DDP. Furthermore, government websites had to be consulted to understand the difference between Delta Decisions, region-specific preferred strategies, and local projects.	N/A
2. Legal sunsets	The DDP explicitly states that strategies and measures can be adapted to new developments, illustrating revisions after specific time periods. ‘The choice was made for an adaptive approach: new developments and insights can be a reason to adjust previously established preferred strategies and (delta) decisions. This can be done every year if required by new developments. The steering group of the delta plan decided in 2017 to also carry out a systematic reassessment every six years.’^78^	The MDP explicitly distinguishes between short-, mid-and long-term measures to be implemented. This ensures that long-term strategies can be left relatively open, so that they can be adapted if socio-ecological conditions change. ‘A primary focus is given to no-regret and priority measures that should be taken in the short- to mid-term (2050). […] For the mid-to long-term (2100), additional measures are presented that are specifically designed to prepare the delta to cope with, and adapt to, the more extreme impacts of climate change.’^79^ This aims to ensure flexible adaptation to unforeseen events.
3. Legally binding authority	The DDP is legally grounded in the Dutch Delta Act and the Water Act. These laws legitimise the decision-making latitude of stakeholders involved in the implementation of the policies and strategies proposed in the DDP. Although the Delta Act and Water Act are mentioned in the DDP, the laws had to be consulted to find out which governmental levels and stakeholders are allocated the authority to make decisions and implement solutions. The principle is therefore implicitly present.	Legally binding authority is illustrated in the MDP by the recommendation to establish a legally mandated entity, the Mekong Delta Planning Commission, that should have sufficient decision-making latitude to manage land and water issues effectively and sustainably in the Mekong delta. The document proposes to institutionalise the authority of the Commission in binding laws. Legally binding authority is therefore explicitly present in the MDP.
4. Legally binding responsibility	The DDP, on multiple occasions, explicitly discusses responsibilities of different stakeholders involved in the DDP and refers to the Water Act where responsibilities are further stipulated.	The MDP explicitly provides illustrations of laws that define and assign responsibilities to various stakeholders, e.g., the Law on Water Resources, which assigns responsibilities regarding water resource management.
5. Tangible support	Multiple explicit references to tangible support are found in the DDP. For example, the document explains that the DDP is financially supported by the Delta Fund, which aims to fund measures and strategies essential for protecting the Netherlands against flooding and water scarcity. ‘The Delta Fund holds the financial resources to finance investments in water safety, freshwater, and water quality […]. A subsidy can be granted from the Delta Fund to finance measures for water safety, freshwater, and water quality for other governmental authorities.’^80^ This illustrates how regional and local governments can be financially supported. Furthermore, an amendment to the Water Act in 2019 ‘makes it possible to provide financial contributions from the Delta Fund to decentralised authorities for taking measures to tackle flood risks.’^81^	The MDP provides multiple explicit references to tangible support. Similar to the DDP, most illustrations of tangible support are in the form of funds. For example, it is argued that the ‘predominantly rural economy of the delta has been well established and developed over the last three decades, primarily as a result of the dedicated investment and support by the Government of Vietnam’.^82^ To stimulate further development of the delta, an agriculture development fund is proposed. It is argued that the Vietnamese government should be active and supportive in stimulating development ‘by investing in, and providing for, direct services – notably in research and development, state operated breeding and hatcheries, and trade regulation and certification support services’,^83^ which needs to be combined with ‘investments in favourable infrastructural developments, in particular waterways and management, that account for sustainable water quality intake, disposal and treatment requirements […] as well as transport and energy services’.^84^
INSTITUTIONAL DESIGN PRINCIPLES	DUTCH DELTA PROGRAMME (DDP)	MEKONG DELTA PLAN (MDP)
1. Well-defined boundaries	The DDP frequently and explicitly provides illustrations of well-defined boundaries. For example, the international character of the Rhine, Meuse and Scheldt rivers is acknowledged in the DDP, and compacts or agreements have been made to govern these international river systems more effectively. For example, ‘Flanders and the Netherlands work together in the Flemish-Dutch Scheldt Commission on an agenda for the future’^85^ and ‘the Netherlands and North Rhine-Westphalia have conducted research together in the Working Group High Water on flood risks in the border area’.^86^ Furthermore, to ‘improve flood risk management and coordinate efforts’ the Netherlands has been divided into 25 safety regions which clarifies the jurisdiction of each region.^87^	The principle of well-defined boundaries is explicitly present in the MDP. For example, chapter 7 of the MDP proposes land and water management measures. Some of these measures are proposed for the entire delta, others specifically for the upper, middle and lower delta regions. It is underlined that measures ‘for flood control, securing adequate freshwater supplies in the dry season, salinity intrusion, regulation, and management of an adequate and healthy brackish water zone for aquaculture, coastal defense, etc. are all typically measures that need to be considered at the delta level, but in their impact and influences they go beyond the boundaries of local governance and policy jurisdiction’.^88^ This illustrates a recognition that measures transcend the local level and that measures taken in one part of the delta impact other areas too. Furthermore, the MDP also calls for an international organisation to stimulate collaboration with upstream countries, as upstream developments have significant impacts on the downstream Mekong delta. ‘Institutional arrangements that facilitate cross-border decision-making and true integration of planning and measures’^89^ are therefore required.
2. Participatory decision-making	It is explicitly mentioned that the DDP is the product of the collaboration between stakeholders. Although the DDP is a national programme, ‘the central government, provinces, municipalities, and regional water authorities work together in an innovative way, based on input from civil society organisations, knowledge institutes, citizens, and businesses.’^90^ The ambition is, where possible, to stimulate ‘the participation of local governments, businesses, and citizens in the preparation of plans and measures’.^91^ The DDP distinguishes between five levels of stakeholder participation, including ‘informing, consulting, advising, co-producing, and (co-)decision-making’.^92^ Additional examples of participatory decision-making can be found in the DDP relating to the implementation of specific projects.	The MDP includes explicit references to participatory decision-making. In the preparatory phase of establishing the MDP, various knowledge institutes were included. In the process of formulating the actual strategies to be included in the MDP, a number of stakeholders and experts, regional and national were involved. These include ‘experts and specialists from different sectors […], decision-makers of local, provincial, and national authorities, [and] representatives from organisations for, e.g., industry, fishery, transport, agri- and aquaculture.^93^ Additionally, the MDP underlines that ‘international organisations like the World Bank, ADB, UNDP and different non-governmental organisations are stakeholders in the sense that they have a good understanding of integrated development and are capable of influencing projects in the delta in conformity with a delta plan approach.^94^
3. Internal enforcement	The DDP explicitly states that stakeholders are obliged to report on their progress to higher authorities. For example, the Netherlands is divided into 42 working regions to realise the ambitions formulated in the plan for spatial adaptation. These working regions ‘monitor the progress in their area and report on the progress. Based on this, the bodies of consultation report progress made back to the delta commissioner’.^95^	N/A
4. Internal conflict resolution	An explicit example is provided of internal conflict resolution related to a specific project for water safety. Within this project, several guiding principles were established. One of these principles is transparency, i.e., ‘we are open to each other; if our individual interest conflicts with the collective interest, we further discuss it’.^96^ Another principle focuses on predictability, i.e., ‘we discuss risks and issues at an early stage, so that we can consider them and make deliberate decisions’.^97^ Lastly, reliability is underlined, i.e., ‘we make clear agreements with each other and honour them’.^98^ This illustrates how agreements are made that stimulate open communication to resolve disputes, and with which stakeholders should comply.	Internal conflict resolution is implicitly present in the MDP. The MDP underlines the importance of equal access to information as a way to reduce conflict. Joint fact-finding is stimulated, which requires the establishment of one team, consisting of experts and decision-makers representing all relevant stakeholders in the Mekong delta, that gathers relevant information about the delta. By gathering information in one place and including representatives from different stakeholder groups, this team would be well equipped to resolve disputes, and this may ensure that ‘relevant authorities become more capable to effectively manage, operate, maintain and enforce rules and policies for land and water management in the Mekong delta’.^99^ This could function as a mechanism of internal conflict resolution.
